# Associations Between Structural Phenotype and Polygenic Risk Scores in Intermediate Age-Related Macular Degeneration – A MACUSTAR Report

**DOI:** 10.1167/tvst.14.9.37

**Published:** 2025-09-26

**Authors:** Lukas Schloesser, Jan H. Terheyden, Charlotte Behning, Hannah Klinkhammer, Davide Garzone, Marlene Saßmannshausen, Sarah Thiele, Steffen Schmitz-Valckenberg, Carel Hoyng, Clara I. Sánchez, Matthias Schmid, Ulrich F. O. Luhmann, Heather Floyd, Sergio Leal, Frank G. Holz, Robert P. Finger, on behalf of the MACUSTAR Consortium

**Affiliations:** 1 Department of Ophthalmology, University of Bonn, Bonn, Germany; 2 Department of Ophthalmology, Medical Faculty Mannheim, Heidelberg University, Mannheim, Germany; 3 Department of Medical Biometry, Informatics and Epidemiology, University of Bonn, Bonn, Germany; 4 Institute for Medical Biometry and Statistics, Medical Faculty, Phillips-University Marburg, Marburg, Germany; 5 Institute for Genomic Statistics and Bioinformatics, University of Bonn, Bonn, Germany; 6 Department of Ophthalmology, University Medical Center – Hamburg-Eppendorf, Hamburg, Germany; 7 Department of Ophthalmology and Visual Sciences, John A. Moran Eye Center, University of Utah, Salt Lake City, UT, USA; 8 RUMC Nijmegen, Nijmegen, The Netherlands; 9 Informatics Institute, University of Amsterdam, Amsterdam, The Netherlands; 10 Roche Pharmaceutical Research and Early Development, Translational Medicine Ophthalmology, Roche Innovation Center, Basel, Switzerland; 11 Novartis Pharma AG, Basel, Switzerland; 12 Bayer Consumer Care AG, Basel, Switzerland

**Keywords:** age-related macular degeneration (AMD), polygenic risk score (PRS), imaging biomarkers, pathogenesis

## Abstract

**Purpose:**

The purpose of this study was to analyze genotype-phenotype associations in intermediate age-related macular degeneration (iAMD) based on global and pathway-specific polygenic risk scores (psPRS) in participants of the prospective European multicenter cohort study MACUSTAR.

**Methods:**

Assessed structural biomarkers included reticular pseudodrusen (RPD), pigmentary abnormalities, hyper-reflective foci (HRF), and incomplete or complete retinal pigment epithelium (RPE) and outer retinal atrophy (iRORA and cRORA). Blood samples were genotyped and imputed via a local pipeline. Global and pathway-specific PRS (complement PRS [C-PRS], with and without *ARMS2*/*HTRA1* variants [C+AH-PRS and AH-PRS]; extracellular matrix PRS [E-PRS]; and lipid PRS [L-PRS]) were calculated. The associations between global and pathway-specific PRS and structural iAMD biomarkers were assessed with multivariable models, controlling for age and sex.

**Results:**

In total, 404 participants (263 women, 65.1%; mean age = 71.5 ± 7.0 years, mean ± standard deviation [SD]) were included in the analysis. Multivariable regression models revealed that RPD was associated with a higher AH-PRS (estimate = 7.11 × 10^−2^, *P* = 9.0 × 10^−3^), C+AH-PRS (estimate = 9.96 × 10^−2^, *P* = 5.0 × 10^−3^), and E-PRS (estimate = 3.28 × 10^−2^, *P* = 3.1 × 10^−2^). The presence of cRORA was associated with a higher AH-PRS (estimate = 1.34 × 10^−1^, *P* = 2 × 10^−3^) and a higher C+AH-PRS (estimate = 1.59 × 10^−1^, *P* = 6 × 10^−3^).

**Conclusions:**

Structural risk biomarkers are associated with psPRS in iAMD. These findings further underscore the heterogeneity of pathogenic pathways in AMD and indicate differential risk characteristics across the broad spectrum of iAMD.

**Translational Relevance:**

Our findings reveal subgroups in iAMD based on genotype-phenotype associations which can help identifying patients at high risk for iAMD and establish new endpoints for clinical trials in iAMD.

## Introduction

Age-related macular degeneration (AMD) is the leading cause of blindness in the industrial world and worldwide in the group of people >55 years of age.^1^^,^^2^ Its pathophysiology is complex and involves a reduced availability of nutrients and oxygen for the outer retina, release of growth factors, chronic inflammation with increased secretion of proinflammatory cytokines (both locally and systemically), and dysregulated innate immunological processes like excessive complement activation.^2^^–^^7^ Additional modifiable and nonmodifiable risk factors, like aging, smoking, and genetics, impact AMD age of onset and progression.^2^

The structural hallmark lesions of AMD are drusen, which are accumulations of extracellular debris underneath the retinal pigment epithelium (RPE).^2^ Accordingly, the clinical classification system by Ferris et al. uses drusen size and the presence of AMD-characteristic pigmentary abnormalities as structural parameters to define early and intermediate stages and the presence of geographic atrophy and choroidal neovascularization to define advanced AMD.^8^ Besides sub-RPE drusen, there is a plethora of other structural features that clinically appear in individuals with AMD but do not sufficiently explain the variable natural history of early AMD stages.^2^ However, recent genetic studies have shown that an individual's genetic background explains up to 70% of the variability of AMD onset, which thus might also contribute to the distinction between AMD sub-phenotypes.^9^^,^^10^

Recent studies investigating genotype-phenotype associations have already helped in defining AMD subtypes by showing that specific structural features, such as reticular pseudodrusen (RPD) and complete RPE and outer retinal atrophy (cRORA) are associated with a higher genetic susceptibility when assessed using a polygenic risk score (PRS) combining known AMD-associated single nucleotide polymorphisms (SNPs).^11^^,^^12^ Additional studies demonstrated AMD subtypes with distinct genetic profiles, which show differing rates of disease progression.^13^^,^^14^ Thus, Schmitz-Valckenberg et al. showed that patients with AMD homozygous for AMD-risk variants in the *ARMS2*/*HTRA1* locus with or without additional risk variants in the *CFH-CFHR5* locus had a higher risk for progression to late AMD as compared with AMD subjects homozygous for risk variants at *CHF-CFHR5* only.^13^ SNPs in the *ARMS2*/*HTRA1* or *CFH* loci are also the ones with the highest effect size among all AMD-associated SNPs.^15^^,^^16^ Nevertheless, combining the known AMD-associated SNPs in global or pathway-specific PRS has the advantage of taking into account the complex genetics of AMD reflected by their higher ability of disease prediction compared with single SNPs.^15^

Recently, the EYE-RISK Consortium has established total and pathway-specific (complement, lipid, and extracellular matrix) PRS,^17^ as well as a score based on *ARMS2* variants.^10^ However, currently, there are no studies comprehensively investigating genotype-phenotype associations among several different structural biomarkers in intermediate AMD (iAMD) and these newly available PRS (global and pathway-specific).^12^^,^^18^ Thus, we investigated the association between various structural features and the above-mentioned total and pathway-specific PRS in the MACUSTAR study. This study is intended to broaden the understanding of genotype-phenotype associations in iAMD and could thus help to identify new subtypes of iAMD based on genotype and phenotype.

## Methods

### MACUSTAR Study

The MACUSTAR study is a prospective multicenter cohort study conducted at 20 clinical sites across 7 different European countries (ClinicalTrials.gov identifier: NCT03349801).^19^ The study focuses on the development and validation of structural, functional, and patient-reported endpoints for patients with iAMD, which can then be used for future clinical trials.^20^ The study assessments are highly standardized across all sites, implemented by trained study personnel, and quality controlled. Assessments include state-of-the-art multi-modal retinal imaging, chart-based and device-based functional assessments, and patient-reported outcomes, as well as optional genetic testing and biobanking. The MACUSTAR study recruited individuals of all AMD stages and healthy control participants, as described previously.^20^ In brief, individuals with early AMD (early AMD in both eyes, *n* = 34) and iAMD (iAMD in at least one eye, *n* = 586) are currently being followed for up to 6 years on a 6- to 12-month basis, and individuals with late AMD (bilateral late AMD, *n* = 43) and healthy control participants (no AMD in either eye, *n* = 56) undertook test-retest assessments early on in the study. Individuals with any eye or systemic conditions (media opacity and eye movement disorder, [i.e. nystagmus and extraocular motility restriction], severe ptosis, head tremor, and chronic ocular disorders [i.e. glaucoma and cataract]) likely to require surgery in the near future, uncontrolled glaucoma, diabetic retinopathy, epiretinal membrane, retinal changes due to other causes than AMD, systemic illness that compromises study participation, participation in any other interventional trial, fluorescein allergy, and cognitive impairment) reducing the interpretability of the outcome measures were excluded.^20^^,^^21^ The MACUSTAR study fulfills all provisions set out in the Declaration of Helsinki, and all included participants provided informed consent before study recruitment and data collection.^20^

### Retinal Imaging and Grading Strategy

Detailed imaging protocols and the consecutive grading strategy can be found in previous publications.^19^^,^^20^^,^^22^

Participants underwent multimodal imaging, spectral domain optical coherence tomography (SD-OCT) using the Spectralis Heidelberg Retina Angiograph (HRA) + OCT device (Heidelberg Engineering, Heidelberg, Germany, digital imaging resolution 768 × 768 pixels) and color fundus photography (CFP).^22^ The Spectralis HRA + OCT device generates an infrared reflectance picture (IR = 30 degrees × 30 degrees, automatic real-time mode [ART] mode × 30 frames) and a set of SD-OCT scans (enhanced depth-imaging mode, centered on the fovea, 30 degrees × 25 degrees, 241 B scans, distance between scans 30 mm, ART mode, 9 frames).^22^

The generated imaging data were transferred to a central reading center (GRADE Reading Center, University of Bonn) and underwent further quality control steps.^22^ Then, the imaging data was graded by two junior graders following a detailed and standardized grading protocol.^22^ In case of a discrepancy between the junior graders, the final grading decision was made by a senior grader.^22^ Junior graders had to complete a structured training program for assessment of retinal imaging in AMD and were required to have prior experience in at least one clinical trial at a reading center.^19^ Senior graders had to be certified board members in ophthalmology with 2 or more years of experience in the assessment of retinal imaging in clinical AMD trials, experience as a grader in at least one clinical trial at the GRADE Reading Center, and undergone standard training and certification by the GRADE Reading Center.^19^

### Qualitative Assessment of Structural Parameters

AMD stages were classified according to Ferris et al. based on CFP and SD-OCT images. Early AMD was defined as the presence of ≥1 medium-sized druse (>63 µm and ≤125 µm) without any pigmentary abnormalities,^8^ and iAMD was defined by the presence of drusen larger than 125 µm. Patients with uni- or bilateral geographic atrophy (GA; defined as an area ≥0.1 mm^2^ in fundus auto-fluorescence [FAF] imaging) or macular neovascularization (MNV) were classified as late AMD. If both eyes met the inclusion criteria of the study groups according to the respective AMD stages, the study eye was the one with the better visual acuity to enhance the reliability of functional testing and the quality of image acquisition, thereby increasing sensitivity in detecting changes over time.^20^

The structural biomarkers that have been included in the analyses are RPD, pigmentary abnormalities (PAs), hyper-reflective foci (HRF), incomplete RPE, and outer retinal atrophy (iRORA) and cRORA.^23^^,^^24^ PAs are defined as the presence of any AMD-associated pigmentary changes.^25^ HRF are hyper-reflective lesions with a reflectivity comparable to the RPE layer, detached from the same, in proximity to drusen, and with a thickness of at least one-third of the RPE/Bruch's membrane (BM) complex.^26^^,^^27^ Reticular pseudodrusen are defined as lesions with a diameter of approximately 100 µm in IR or FAF imaging and correspond to hyper-reflective abnormalities/elevations above the RPE/BM complex in SD-OCT imaging.^28^^,^^29^ The presence was confirmed when at least five of those lesions were present in more than one B-scan. The cRORA lesions had to fulfill the following 4 criteria in SD-OCT imaging: choroidal hypertransmission over a distance of 250 µm, disruption or attenuation of RPE over a distance of 250 µm, combined photoreceptor degeneration in this area, and absence of any RPE tear.^24^ The iRORA lesions had to fulfill the criteria except for the distance criteria of 250 µm.^23^ The iRORA and cRORA were calculated in a combined model due to sample size limitations with the iRORA group encompassing patients with at least one iRORA lesion but not one cRORA lesion and the cRORA group encompassing patients with at least one cRORA lesion.

### Genotyping

Specific consent for genetic testing was obtained from participants of the MACUSTAR study. In the case of consent, blood samples were collected during the screening visit at baseline (V1). Blood samples were then genotyped with the modified Illumina Infinium GSA Array (Illumina, San Diego, CA; in collaboration with the Synergy RPD Consortium) and imputed via a local pipeline comparable to the Michigan Imputation Server. SNPs with an imputation quality of *R*^2^ < 0.3 were discarded. Further quality controls included exclusion of SNPs with minor allele frequency of less than 1%, missing genotype call rate of more than 10%, or violating the Hardy-Weinberg equilibrium (*P* < 10^–6^). Genotype data were processed using Plink-Software (version 2.00, developed by Christopher Chang and Shaun Purcell, Cambridge, MA).^18^

### Polygenic Risk Scores Design and Calculation

The overall AMD PRS was calculated based on beta estimates of 69 risk loci, reported in a meta-analysis by Han et al. in 2020.^18^ The MACUSTAR study was not a part of any genome-wide association study (GWAS) that identified the AMD-associated SNPs used in this analysis. After quality control, 50 of those 69 SNPs were available for our cohort. The exact calculation of the PRS was performed according to the publication of Lambert et al. in 2021^30^ and PRS are reported as non-averaged score sums. For further differentiation of genetic risk profiles regarding the underlying pathophysiologic processes, pathway-specific PRS (psPRS; complement-specific PRS [C-PRS]; *ARMS2/HTRA1* [AH-PRS]; combined complement and *ARMS2/HTRA1* [C+AH-PRS]; extracellular matrix-specific [E-PRS]; and lipid-specific [L-PRS]) were calculated as described by Colijn et al. (2020) and a score based on the *ARMS2* variant developed by Biarnes et al. (2020).^10^^,^^17^ The psPRS are based on the grouping of risk loci according to the biological process the respective gene product is involved in.^31^ As the subdivision into psPRS by Colijn et al. was based on an older GWAS meta-analysis by Fritsche et al. (2013), we checked if the SNPs of the 12 newly identified ones by Han et al. compared to the GWAS meta-analysis by Fritsche et al. could be assigned to one of the psPRS.^17^^,^^18^^,^^32^ One of those SNPs is located in the *ADAM19* locus, which encodes for a matrix metalloproteinase involved in extracellular matrix regulation and in a broader sense regulation of inflammation by cleavage immunological mediators like specific cytokines.^33^ A second previously known AMD-associated SNP is associated with *MMP9* locus, which encodes also for a matrix metalloproteinase that regulates tissue remodeling by activation of cytokines and chemokines by cleavage.^34^ As these two SNPs can be linked to functions in regulation of extracellular matrix, we included them in the E-PRS (Table 1). There are slight differences between the composition of the psPRS used in this study and the ones established in the publication of Colijn et al. (2020) due to a lack of specific SNPs on the genotyping array used for the MACUSTAR study (e.g. complement-associated SNPs in the *TMEM97/VTN* and *C9* loci).^17^

**Table 1.** Construction of Pathway-Specific Polygenic Risk Scores

Assumed Pathway	Assigned Genes of the Respective SNPs Included for the Respective psPRS
Complement	*C3*, *CFB/C2*, *CFH*, *CFI*
ECM	***ADAM19***, *ADAMTS9-AS2*, *COL4A3*, *COL8A1*, ***MMP9***, *SYN3/TIMP3*, *VEGF-A*
Lipid metabolism	*ABCA1*, *APOE*, *CETP*, *LIPC*
ARMS2/HTRA1	*ARMS2/HTRA1*

ECM, extracellular matrix.

### Statistical Analysis

All statistical calculations were carried out using the statistics software program R (version 4.3.0.; R Foundation for Statistical Computing, Vienna, Austria). Baseline characteristics were reported with mean (± standard deviation [SD]) and median (with minimum and maximum) for continuous variables and numbers with percentages for binary variables (Table 2). Multivariable linear regression models adjusted for sex and age were fitted to investigate the associations between the presence of one or more structural biomarkers and the PRS. The regression models were constructed with the structural biomarkers (as well as sex and age) as independent variables and with specific PRS (global and pathway-specific PRS). Any *P* < 0.05 was considered significant. The *P* values are reported as part of an exploratory analyses; accordingly, they were not adjusted for multiple testing.

**Table 2.** Participant Characteristics and Polygenic Risk Scores

	No AMD (*n* = 46)	Early AMD (*n* = 33)	Intermediate AMD (*n* = 404)	Late AMD (*n* = 73)	Overall (*n* = 556)
Age
Mean ± SD	67.9 ± 6.24	71.4 ± 6.26	71.5 ± 6.98	74.7 ± 6.19	71.6 ± 6.94
Median [min, max]	68.0 [56.0, 80.0]	72.0 [57.0, 82.0]	72.0 [55.0, 86.0]	76.0 [60.0–84.0]	72.0 [55.0, 86.0]
Sex					
Female	28 (60.9%)	26 (78.8%)	263 (65.1%)	44 (60.3%)	361 (64.9%)
Male	18 (39.1%)	7 (21.2%)	141 (34.9%)	29 (39.7%)	195 (35.1%)
Study eyes					
Right, *n*	26 (56.5%)	13 (39.4%)	205 (50.7%)	37 (50.7%)	281 (50.5%)
Left, *n*	20 (43.5%)	20 (60.6%)	199 (49.3 %)	36 (49.3 %)	275 (49.5%)
BCVA, logMAR					
Mean ± SD	−3.61 × 10^−2^ ± 8.20 × 10^−2^	1.45 × 10^−2^ ± 8.14 × 10^−2^	2.23 × 10^−2^ ± 1.04 × 10^−1^	4.05 × 10^−1^ ± 4.02 × 10^−1^	6.74 × 10^−2^ ± 2.18 × 10^−1^
Median [min, max]	−5.00 × 10^−2^	2.00 × 10^−2^	2.00 × 10^−2^	2.40 × 10^−1^	2.00 × 10^−2^
	[−1.60 × 10^−1^, 1.40 × 10^−2^]	[−1.80 × 10^−1^, 2.00 × 10^−1^]	[−2.40 × 10^−1^, 2.80 × 10^−1^]	[−1,00 × 10^−1^, 1.24]	[−2.40 × 10^−1^, 1.24]
PRS, mean ± SD					
Global PRS	−2.07 × 10^−1^ ± 5.57 × 10^−1^	−4.74 × 10^−2^ ± 4.17 × 10^−1^	1.87 × 10^−1^ ± 4.25 × 10^−1^	2.58 × 10^−1^ ± 3.82 × 10^−1^	n/a
AH-PRS	3.54 × 10^−2^ ± 1.93 × 10^−1^	8.97 × 10^−2^ ± 1.98 × 10^−1^	1.60 × 10^−2^ ± 2.26 × 10^−1^	2.06 × 10^−1^ ± 2.38 × 10^−1^	n/a
C+AH-PRS	−2.72 × 10^−1^ ± 3.28 × 10^−1^	−2.22 × 10^−1^ ± 3.19 × 10^−1^	−3.70 × 10^−2^ ± 2.96 × 10^−1^	1.80 × 10^−3^ ± 2.93 × 10^−1^	n/a
C-PRS	−3.08 × 10^−1^ ± 2.35 × 10^−1^	−3.12 × 10^−1^ ± 2.29 × 10^−1^	−1.97 × 10^−1^ ± 1.83 × 10^−1^	−2.04 × 10^−1^ ±1.74 × 10^−1^	n/a
E-PRS	5.24 × 10^−2^ ± 1.37 × 10^−1^	1.21 × 10^−1^ ± 1.15 × 10^−1^	1.03 × 10^−1^ ± 1.26 × 10^−1^	1.28 × 10^−1^ ± 1.06 × 10^−1^	n/a
L-PRS	3.01 × 10^−3^ ± 1.38 × 10^−1^	8.48 × 10^−3^ ± 9.01 × 10^−2^	2.74 × 10^−2^ ± 1.10 × 10^−1^	1.86 × 10^−2^ ± 1.02 × 10^−1^	n/a

AH-PRS, ARMS2/HRTA1-PRS; AMD, age-related macular degeneration; ARMS2, age-related maculopathy susceptibility protein 2; BCVA, best-corrected visual acuity; C+AH-PRS, complement/ARMS2/HTRA1-PRS; C-PRS, complement-PRS; E-PRS, extracellular matrix-PRS; HTRA1, serine protease HTRA; L-PRS, lipid-PRS; logMAR, logarithm of the minimum angle of resolution; PRS, polygenic risk score; SD, standard deviation.

## Results

### Baseline Characteristics and Stage-Dependent PRS

From the baseline visit of the longitudinal part of the MACUSTAR study, in total, 556 participants with absent features of AMD (*n* = 46), early AMD (*n* = 33), bilateral intermediate AMD (*n* = 404), and late AMD (*n* = 73) were included in the analyses. The mean age was 71.4 ± 6.26 years with 21.2% male participants (*n* = 7) in the early AMD group, 71.5 ± 6.98 years with 34.9% male participants (*n* = 141) in the intermediate AMD group, 74.7 ± 6.19 years with 39.7% male participants (*n* = 29) in the late AMD group, and 67.9 ± 6.24 years with 39.1% male participants (*n* = 18) in the no AMD group. The average logarithm of the minimum angle of resolution (logMAR) of best corrected visual acuity (BCVA) was 6.74 × 10^−2^ ± 2.18 × 10^−1^, with 1.45 × 10^−2^ ± 8.14 × 10^−2^ in the early AMD group, 2.23 × 10^−2^ ± 1.04 × 10^−1^ in the intermediate AMD group, 4.05 × 10^−1^ ± 4.02 × 10^−1^ in the late AMD group, and −3.61 × 10^−2^ ± 8.20 × 10^−2^ in the no AMD group. The PRS including all relevant quality controlled SNPs based on Han et al. (2020) was −2.07 × 10^−1^ ± 5.57 × 10^−1^ (mean ± SD) in the group without AMD, −4.74 × 10^−2^ ± 4.17 × 10^−1^ in the early AMD group, 1.87 × 10^−1^ ± 4.25 × 10^−1^ in the intermediate AMD group, and 2.58 × 10^−1^ ± 3.82 × 10^−1^ in the late AMD group.^18^ Table 2 provides detailed information regarding baseline characteristics and the stage-dependent PRS.

### Qualitative Assessment of Structural Parameters in iAMD

In the bilateral intermediate AMD group (*n* = 404) the presence of different structural parameters, other than stage-defining large drusen, was assessed. Presence of PA was detected in 47.8% of the eyes, followed by HRF in 46.8%, PRD in 23.8%, iRORA in 8.7%, and cRORA in 7.2% of the eyes (Table 3).

**Table 3.** Prevalence of Structural Parameters in Patients With iAMD

Structural Parameter in the Study Eye	iAMD (*n* = 404) *n*
RPD	96 (23.8%)
PA	193 (47.8%)
HRF	189 (46.8%)
iRORA	35 (8.7%)
cRORA	29 (7.2%)

HRF, hyper-reflective foci; i/cRORA, incomplete/complete retinal pigment epithelium and outer retinal atrophy; iAMD, intermediate age-related macular degeneration; PA, pigmentary abnormalities; RPD, reticular pseudodrusen.

### Association Between Global PRS and AMD Stage

In multivariable linear regression analyses comparing PRS across AMD stages, controlling for sex and age, (bilateral) iAMD (estimate = 4.05 × 10^−1^, *P* < 1 × 10^−3^) and late AMD (late AMD in at least one eye, estimate = 4.81 × 10^−1^, *P* < 1 × 10^−3^) were associated with higher PRS values as expected (Table 4) compared with participants of the control group. There were no associations for controls and early AMD. Box plots were generated to visualize associations between PRS and AMD stages (see the Fig.). A pairwise comparisons of estimated marginal means indicating the differences in mean global PRS values between AMD stages can be found in Supplementary Table S1. The reported *P* values are not adjusted for multiple comparisons. This analysis also shows a significant difference between global PRS values of iAMD and late AMD compared with patients with early AMD or healthy controls.

**Table 4.** Multivariable Regression Models Including Global PRS (Dependent Variable) and AMD Stages (Independent Variables), Adjusted for Age and Sex

Predictors	Estimate [95% CI]	*P* Value
Early AMD	1.75 × 10^−1^ [−2.05 × 10^−2^ to 3.7 × 10^−1^]	7.9 × 10^−2^
Intermediate AMD	4.05 × 10^−1^ [2.71 × 10^−1^–5.38 × 10^−1^]	**<1.0 × 10^−^^3^**
Late AMD	4.81 × 10^−1^ [3.17 × 10^−1^ to 6.45 × 10^−1^]	**<1.0 × 10^−^^3^**
Age	−2.5 × 10^−3^ [−7,8 × 10^−3^ to 2.9 × 10^−3^]	3.63 × 10^−1^
Sex (male)	3.57 × 10^−2^ [−4.04 × 10^−2^ to 1.12 × 10^−1^]	3.57 × 10^−1^

AMD, age-related macular degeneration.

The *P* values in bold represent statistical significance.

The control group and female sex are reference categories in the analysis.

**Figure.**
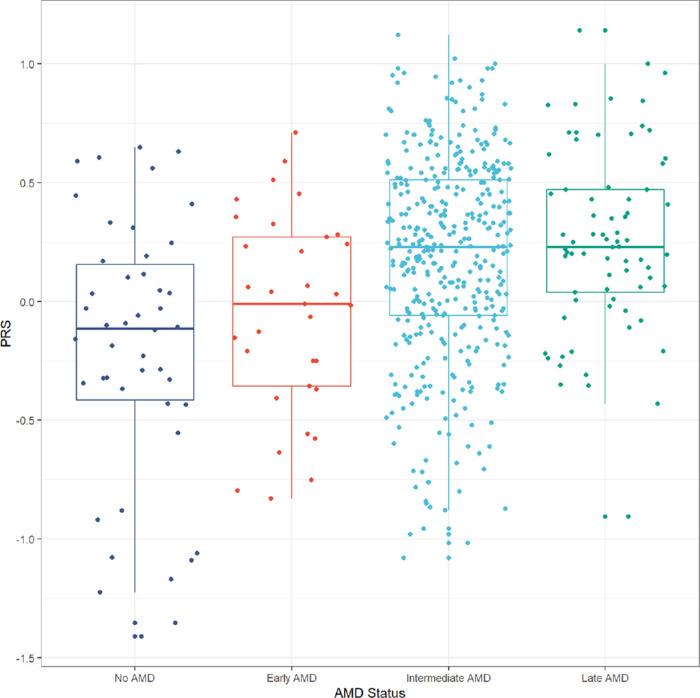
Box plot visualizing the distribution of the global PRS by AMD stage (Beckman classification) and presence of AMD. Every *dot* represents a participant. The *P* values and estimates for marginal effects of differences between AMD stages can be found in Supplementary Table S1.

### Association of Structural Parameters in iAMD With Global PRS

Next, we looked at the differences in PRS according to structural parameters, including RPD, PA, HRF, iRORA, and cRORA in eyes with (bilateral) iAMD. In separate multivariable linear regression models for each structural biomarker controlling for sex and age, RPD (*P* = 1.3 × 10^−2^) and cRORA (*P* = 2.9 × 10^−2^) were associated with higher global PRS values, whereas PA, HRF, and iRORA were not associated (Table 5). Extended multivariable linear regression analyses, including multiple structural parameters, can be reviewed in Supplementary Tables S2 and S3.

**Table 5.** Multivariable Regression Models Including Global PRS (Dependent Variable) and a Specific Structural Biomarker (Independent Variable), Adjusted for Age and Sex

Model/Predictors	Estimate [95% CI]	*P* Value
1 / RPD	1.27 × 10^−1^ [2.67 × 10^−2^ to 2.26 × 10^−2^]	**1.3 × 10^−^^2^**
2 / PA	7.65 × 10^−2^ [−7.3 × 10^−3^ to 1.60 × 10^−1^]	7.3 × 10^−2^
3 / HRF	3.96 × 10^−2^ [−4.41 × 10^−2^ to 1.23 × 10^−1^]	3.53 × 10^−2^
4 / iRORA	−2.56 × 10^−2^ [−1.75 × 10^−1^ to 1.23 × 10^−1^]	7.35 × 10^−2^
4 / cRORA	1.80 × 10^−1^ [1.87 × 10^−2^ to 3.42 × 10^−1^]	**2.9 × 10^−^^2^**

cRORA, complete retinal pigment epithelium and outer retinal atrophy; HRF, hyper-reflective foci; iRORA, incomplete retinal pigment epithelium and outer retinal atrophy; PA, pigmentary abnormalities; RPD, reticular pseudodrusen.

The *P* values in bold represent statistical significance.

### Associations Between Structural Parameters and psPRS

Repeating these multivariable regression analyses for each psPRS, RPD in eyes with bilateral iAMD was associated with higher values of the AH-PRS (*P* = 9.0 × 10^−3^), the C+AH-PRS (*P* = 5.0 × 10^−3^), and the E-PRS (*P* = 3.1 × 10^−2^), and no associations with C-PRS (*P* = 1.97 × 10^−1^) or L-PRS (*P* = 1.07 × 10^−1^) were found. Presence of cRORA was associated with higher values of the AH-PRS (*P* = 2 × 10^−3^) and the C+HA-PRS (*P* = 6 × 10^−3^). No other structural biomarkers were associated with any psPRS (Table 6).

**Table 6.** Multivariable Regression Models With a psPRS (Dependent Variable) and a Specific Structural Biomarker (Independent Variable), Adjusted for Age and Sex

	AH-PRS		C+AH-PRS		C-PRS		E-PRS		L-PRS	
Model/Predictors	Estimate [95% CI]	*P* Value	Estimate [95% CI]	*P* Value	Estimate [95% CI]	*P* Value	Estimate [95% CI]	*P* Value	Estimate [95% CI]	*P* Value
1/RPD	7.11 × 10^−2^ [1.81 × 10^−2^ to 1.24 × 10^−1^]	**9.0 × 10^−^^3^**	9.96 × 10^−2^ [3.02 × 10^−2^ to 1.69 × 10^−1^]	**5.0 × 10^−^^3^**	2.84 × 10^−2^ [−1.49 × 10^−2^ to 7.17 × 10^−2^]	1.97 × 10^−1^	3.28 × 10^−2^ [3.10 × 10^−3^ to 6.25 × 10^−2^]	**3.1 × 10^−^^2^**	−2.13 × 10^−2^ [−4.73 × 10^−2^ to 4.6 × 10^−3^]	1.07 × 10^−1^
2/PA	1.79 × 10^−2^ [−2.68 × 10^−2^ to 6.26 × 10^−2^]	4.32 × 10^−1^	4.25 × 10^−2^ [−1.60 × 10^−2^ to 1.01 × 10^−1^]	1.54 × 10^−1^	2.47 × 10^−2^ [−1.18 × 10^−2^ to 6.11 × 10^−2^]	1.84 × 10^−1^	1.33 × 10^−2^ [−1.19 × 10^−2^ to 3.84 × 10^−2^]	3.00 × 10^−1^	1.77 × 10^−2^ [−3.9 × 10^−3^ to 3.94 × 10^−2^]	1.09 × 10^−1^
3/HRF	−8,4 × 10^−3^ [−5.29 × 10^−2^ to 3.62 × 10^−2^]	7.13 × 10^−1^	1.63 × 10^−2^ [−4.21 × 10^−2^ to 7.46 × 10^−2^]	5.84 × 10^−1^	2.46 × 10^−2^ [−1.14 × 10^−2^ to 6.07 × 10^−2^]	1.80 × 10^−1^	6.4 × 10^−3^ [−1.85 × 10^−2^ to 3.13 × 10^−2^]	6.14 × 10^−1^	−5.8 × 10^−3^ [−2.75 × 10^−2^ to 1.59 × 10^−2^]	5.98 × 10^−2^
4/iRORA	−5.49 × 10^−2^ [−1.33 × 10^−1^ to 2.35 × 10^−2^]	1.70 × 10^−2^	−7.51 × 10^−2^ [−1.78 1.59 × 10^−1^ to 2.80 × 10^−2^]	1.53 × 10^−1^	−2.02 × 10^−2^ [−8.48 × 10^−2^ to 4.44 × 10^−2^]	5.4 × 10^−1^	3.10 × 10^−2^ [−1.34 × 10^−2^ to 7.54 × 10^−2^]	1.71 × 10^−1^	8 × 10^−4^ [−3.80 × 10^−2^ to 3.95 × 10^−2^]	9.69 × 10^−1^
4/cRORA	1.34 × 10^−1^ [4.84 × 10^−2^ to 2.19 × 10^−1^]	**2 × 10^−^^3^**	1.59 × 10^−1^ [4.67 × 10^−2^ to 2.70 × 10^−1^]	**6 × 10^−^^3^**	2.50 × 10^−2^ [−4.52 × 10^−2^ to 9.51 × 10^−2^]	4.84 × 10^−1^	−3.1 × 10^−3^ [−5.13 × 10^−2^ to 4.51 × 10^−2^]	9 × 10^−1^	−9.4 × 10^−3^ [−5.14 × 10^−2^ to 3.27 × 10^−2^]	6.62 × 10^−1^

AH-PRS, ARMS2/HRTA1-PRS; ARMS2, age-related maculopathy susceptibility protein 2; C-PRS, complement-PRS; C+AH-PRS, complement/ARMS2/HTRA1-PRS; cRORA, complete retinal pigment epithelium and outer retinal atrophy; E-PRS, extracellular matrix-PRS; HRF, hyper-reflective foci; HTRA1, serine protease HTRA; iRORA, incomplete retinal pigment epithelium and outer retinal atrophy; L-PRS, lipid-PRS; PA, pigmentary abnormalities; PRS, polygenic risk score; RPD, reticular pseudodrusen.

The *P* values in bold represent statistical significance.

## Discussion

In the MACUSTAR study, RPD and cRORA were associated with AMD PRS as well as several pathway-specific genetic risk scores. Whereas our study was able to replicate the strong association between high-risk variants of the *ARMS2/HTRA1* gene and structural biomarkers, our study adds associations between genes of the ECM and RPD to the literature. We found associations of the presence of RPD and cRORA with higher global PRS values in multivariable linear regression models adjusted for sex and age. The association of RPD with higher global PRS values was even found in an extended multivariable regression model, including other structural biomarkers like PA and HRF, although it was lost when iRORA and cRORA were also included.

There are several studies investigating genotype-phenotype correlations in AMD. In the MACUSTAR study, the identified associations of RPDs with AH-PRS and C+AH-PRS seem to predominantly depend on *ARMS2*/*HTRA1* SNPs contained in both psPRS, as the C-PRS is not associated with any of the investigated structural biomarkers. The same constellation was found for cRORA, which is associated with AH-PRS and C+AH-PRS but not with C-PRS. PA, HRF, and iRORA revealed no associations with any PRS. These findings support the status of RPD and cRORA as high-risk structural biomarkers that are associated with accelerated disease progression.^35^^,^^36^

The association of RPD with the latest E-PRS (based on Han et al.) in our analyses is a novel finding, although the association with an older E-PRS based on Fritsche et al. and with *VEGFA* risk variants, which is part of the E-PRS in our study, has already been reported.^12^^,^^18^^,^^32^^,^^37^ There are speculations that the association between *VEGFA* and RPD might underline the connection between RPD and observed choroidal changes, including choroidal thinning or the orientation of RPD along watershed zones of the choroid.^38^^,^^39^ Nevertheless, the pathophysiologic significance of this association remains unclear.

Previous studies already found higher values of global PRS to be associated with different structural biomarkers in intermediate AMD, such as RPD, drusen and large drusen area, and iRORA, as well as cRORA.^11^^,^^12^ In the case of RPD, a higher underlying genetic susceptibility fits the picture of previous findings with an increased risk of progression to GA as well as choroidal neovascularization (CNV).^14^^,^^40^ In a publication by Biarnes et al. (2020), a subgroup of extrafoveal GA and RPD, which is associated with higher E-PRS and a higher AH-PRS, could be identified.^10^ The other two subgroups of GA identified in this study based on phenotype and genotype were a group with high C-PRS, large soft drusen, and foveal atrophy, and another group with a low global PRS, few drusen (any type), and foveal atrophy.^10^ RPD was also reported as a feature of a subgroup of GA characterized by the presence of RPD in combination with high C-PRS values.^41^ These associations are also in line with our results for cRORA, as cRORA is the direct precursor lesion of GA or even identical if it covers a certain area.^22^^,^^23^ Furthermore, the association of RPD with risk variants of *C3*, *CFH*, and *ARMS2/HTRA1* has been shown previously.^37^^,^^42^ HRFs are not specific for AMD and their structural and pathophysiologic correlate is not yet fully understood.^43^ Therefore, it is also unknown whether HRFs reflect the same pathophysiologic sub-process in the context of the respective diseases in which they can be observed.^43^ Our results regarding the missing association of PA with higher global or psPRS are compatible with the results of the NICOLA study, which also did not find an association with higher global PRS values.^11^ Interestingly, iRORA showed no association with higher global or psPRS, although it is the direct precursor lesion of cRORA. This potential discrepancy regarding the involved pathways associated with psPRS could be due to underlying subgroups with a different temporal profile of progression. Thus, there might be an iRORA subgroup characterized by lower global or psPRS values and slow progression, which could account for the majority of iRORA lesions in the iAMD group.

The main strengths of our study and the MACUSTAR study itself are its cohort size, especially with a large number of patients with iAMD, and the extent of deep systematic phenotyping using multimodal retinal imaging with highest quality standards, including standard operating procedures (SOPs), trained site personnel (for image acquisition), and a standardized grading process. The systematic evaluation of structural parameters in iAMD with global and psPRS in our study provides new insights regarding their underlying differential genetic susceptibility. The PRS approach (global or pathway-specific) has also the advantage that including AMD-associated SNPs significantly improves disease prediction.^15^^,^^44^ Our study underscores the importance of certain structural parameters for the disease onset and potentially indicates an increased risk of disease progression. However, further studies using longitudinal data need to investigate the impact of structural parameters in combination with global and psPRS on disease progression.

The main limitation of studies involving genetics in AMD research is the limited sample size, as the underlying genetic background is complex and the degree of interindividual variability is high. The use of cross-sectional data also implies that no statements about the probability of disease progression depending on the presence of a specific structural parameter or its association with one of the different PRS can be made. Furthermore, we included only structural parameters in our analyses which does not allow any structure-function correlations. However, this aspect could be targeted in future studies using data from the extensive functional assessment of the MACUSTAR study. There are also methodical considerations which need to be discussed. The assignment of an SNP to the corresponding gene locus is based on the proximity of the respective SNP to the closest gene locus. This approach is prone to underestimating the complexity of gene regulation due to tertiary interactions of distant genomic loci with each other. Additionally, the functional consequence for the final gene product is unknown in most cases. Therefore, the grouping of SNPs into pathway-specific PRS needs to be understood as an attempt to structure the heterogenous group of AMD-associated SNPs and to further investigate the influence of specific pathways on the disease phenotype. The choice of the structural biomarker as an independent variable also does not reflect the causal relation of the underlying pathobiology but allows descriptive comparison of PRS in groups with present/absent structural biomarkers. The assumption of causal connections based on these genotype-phenotype associations is speculative and must be judged with caution. Furthermore, in January 2024, a new publication by He et al. identified 65 AMD-associated SNPs including 9 novel SNPs.^16^ The resulting PRS constructed in the frame of this paper slightly outperformed the pre-existing PRS from Han et al.^18^

In conclusion, our study could reproduce an association of RPD and cRORA with higher global PRS values. Furthermore, we could show that the presence of specific structural biomarkers in patients with iAMD is differentially associated with different psPRS which further underscores the genetic heterogenicity among patients with iAMD and their broad phenotypic spectrum. Based on this, psPRS have value in further characterizing the heterogeneous group of patients with iAMD which may aid our understanding of this heterogenous AMD disease stage. All in all, this study adds a valuable contribution to the understanding of genotype-phenotype associations in iAMD. Future studies should focus on the impact of genotype-phenotype associations on progression to late AMD which may help identify patients with a high risk of iAMD.

## Supplementary Material

Supplement 1

## Appendix A. Appendix: The MACUSTAR Consortium

H. Agostini, I. D. Aires, L. Altay, R. Atia, F. Bandello, P. G. Basile, J. Batuca, C. Behning, M. Belmouhand, M. Berger, A. Binns, C. J. F. Boon, M. Böttger, J. E. Brazier, C. Carapezzi, J. Carlton, A. Carneiro, A. Charil, R. Coimbra, D. Cosette, M. Cozzi, D. P. Crabb, J. Cunha-Vaz, C. Dahlke, H. Dunbar, R. P. Finger, E. Fletcher, M. Gutfleisch, F. Hartgers, B. Higgins, J. Hildebrandt, E. Höck, R. Hogg, F. G. Holz, C. B. Hoyng, A. Kilani, J. Krätzschmar, L. Kühlewein, M. Larsen, S. Leal, Y. T. E. Lechanteur, D. Lu, U. F. O. Luhmann, A. Lüning, N. Manivannan, I. Marques, C. Martinho, A. Miliu, K. P. Moll, Z. Mulyukov, M. Paques, B. Parodi, M. Parravano, S. Penas, T. Peters, T. Peto, S. Priglinger, R. Ramamirtham, R. Ribeiro, D. Rowen, G. S. Rubin, J. Sahel, C. Sánchez, O. Sander, M. Saßmannshausen, M. Schmid, S. Schmitz-Valckenberg, J. Siedlecki, R. Silva, E. Souied, G. Staurenghi, J. Tavares, D. J. Taylor, J. H. Terheyden, A. Tufail, P. Valmaggia, M. Varano, A. Wolf, and N. Zakaria.
