# Supplementary material for: Associations Between Structural Phenotype and Polygenic Risk Scores in Intermediate Age-Related Macular Degeneration – A MACUSTAR Report
Source: Transl Vis Sci Technol. 2025 Sep 26;14(9):37. doi: 10.1167/tvst.14.9.37 (PMC12489858; doi:10.1167/tvst.14.9.37)
Supplement: Supplement 1 [file tvst-14-9-37_s001.pdf]

SUPPLEMENTAL MATERIAL

Table S1. Pairwise comparisons of estimated marginal means reflecting mean difference of global PRS values between the different AMD stages

Contrast	Estimate	P-value
No AMD - Early AMD	-1.78×10^{-1}	7.93×10^{-2}
No AMD - Intermediate AMD	-4.08×10^{-1}	**$< 1.0 \times 10^{-3}$**
No AMD - Late AMD	-4.81×10^{-1}	**$< 1.0 \times 10^{-3}$**
Early AMD - Intermediate AMD	-2.30×10^{-1}	**3.5×10^{-3}**
Early AMD - Late AMD	-3.06×10^{-1}	**8×10^{-4}**
Intermediate AMD - Late AMD	-7.63×10^{-2}	1.71×10^{-1}
AMD = Age-related macular degeneration		

Associations of structural parameter and global PRS in extended multivariable regression models

We further investigated if these associations we found between RPD and cRORA and the global PRS remain in extended multivariable linear regression models including RPD, PA, HRF, with and without i/cRORA, adjusting for sex and age. First, we calculated a multivariable linear regression model without iRORA and cRORA showing an association between RPD and the global PRS ($p=4.7 \times 10^{-2}$) despite the presence of PA and HRF (Table S2). When including iRORA and cRORA in this model this association was not found anymore (Table S3).

Table S2. Extended multivariable regression analysis with global PRS (dependent variable) and different structural biomarkers (independent variables) except for iRORA or cRORA, adjusted for age and sex

Predictors	Estimate [95% CI]	P-value
RPD	1.04×10^{-2} [1.4×10^{-3} – 2.07×10^{-1}]	**4.7×10^{-2}**
PA	6.83×10^{-2} [-2.62×10^{-2} – 1.63×10^{-1}]	1.56×10^{-1}
HRF	-1.28×10^{-2} [-1.07×10^{-1} – 8.14×10^{-2}]	7.90×10^{-1}
Age	-7.4×10^{-3} [-1.36×10^{-2} – -1.2×10^{-3}]	**1.9×10^{-2}**
Sex (male)	5.73×10^{-2} [-3.11×10^{-2} – 1.46×10^{-1}]	2.04×10^{-1}
HRF = Hyperreflective foci, PA = Pigmentary abnormalities, RPD = Reticular pseudodrusen		

Table S3. Extended multivariable regression analysis with global PRS (dependent variable) and all structural biomarkers (independent variables), adjusted for age and sex

Predictors	Estimate [95% CI]	P-value
RPD	9.68×10^{-2} [-8.1×10^{-3} – 2.02×10^{-1}]	7.0×10^{-2}
PA	6.50×10^{-2} [-3.03×10^{-2} – 1.60×10^{-1}]	1.81×10^{-1}
HRF	-2.08×10^{-2} [-1.17×10^{-1} – 7.58×10^{-2}]	6.73×10^{-1}
iRORA	-7.66×10^{-2} [-2.31×10^{-1} – 7.75×10^{-2}]	3.29×10^{-1}
cRORA	1.15×10^{-1} [-5.97×10^{-2} – 2.90×10^{-1}]	1.96×10^{-1}
Age	-7.3×10^{-3} [-1.35×10^{-2} – -1.1×10^{-3}]	**2.0×10^{-2}**
Sex (male)	6.18×10^{-2} [-2.70×10^{-2} – 1.51×10^{-1}]	1.72×10^{-1}
iRORA = Incomplete retinal pigment epithelium and outer retinal atrophy, cRORA = Complete retinal pigment epithelium and outer retinal atrophy, HRF = Hyperreflective foci, PA = Pigmentary abnormalities, RPD = Reticular pseudodrusen		

Table S4. Linear mixed-effects models with monocular BCVA as outcome variable and sex, age, structural biomarker presence in the respective eye, and PRS as fixed effects, adjusted for age and sex. A random intercept term is included to account for repeated BCVA measurements in both eyes of a participant. Addionally, we included interaction terms in these models to account for interaction effects between PRS and structural biomarkers.

Model	Predictors	Estimate [95% CI]	P-value
1	Global PRS	6.7×10^{-3} [-2.35×10^{-2} – 3.69×10^{-2}]	6.65×10^{-1}
	RPD	3.04×10^{-2} [-7×10^{-4} – 6.14×10^{-2}]	5.5×10^{-2}
	Global PRS x RPD	2.74×10^{-2} [-3.54×10^{-2}– 9.01×10^{-2}]	3.92×10^{-1}
2	AH-PRS	7.9×10^{-3} [-4.9×10^{-2} – 6.48×10^{-2}]	7.86×10^{-1}
	RPD	1.79×10^{-2} [-1.73×10^{-2} – 5.31×10^{-2}]	3.19×10^{-1}
	AH-PRS x RPD	9.65×10^{-2} [-2.11×10^{-2} – 2.14×10^{-1}]	1.08×10^{-1}
3	C+AH-PRS	1.32×10^{-2} [-2.93×10^{-2} – 5.57×10^{-2}]	5.42×10^{-1}
	RPD	3.49×10^{-2} [7.7×10^{-3} – 6.21×10^{-2}]	**1.2×10^{-2}**
	C+AH-PRS x RPD	6.48×10^{-2} [-3.01×10^{-2} – 1.60×10^{-1}]	1.8×10^{-1}
4	C-PRS	2.23×10^{-2} [-4.6×10^{-2} – 9.07×10^{-2}]	5.22×10^{-1}
	RPD	3.69×10^{-2} [-1.7×10^{-3} – 7.54×10^{-2}]	6.1×10^{-2}
	C-PRS x RPD	-5.2×10^{-3} [-1.59×10^{-1} – 1.48×10^{-1}]	9.47×10^{-1}
5	E-PRS	-7.4×10^{-3} [-1.07×10^{-1} – 9.22×10^{-2}]	8.84×10^{-1}
	RPD	4.86×10^{-2} [1.08×10^{-2} – 8.65×10^{-2}]	**1.2×10^{-2}**
	E-PRS x RPD	-7.98×10^{-2} [-3.03×10^{-1} – 1.44×10^{-1}]	4.83×10^{-1}
6	L-PRS	-3.22×10^{-2} [-1.48×10^{-1} – 8.40×10^{-2}]	5.87×10^{-1}
	RPD	3.52×10^{-2} [8.1×10^{-3} – 6.24×10^{-2}]	**1.1×10^{-2}**
	L-PRS x RPD	2.33×10^{-1} [-5.4×10^{-3} – 4.72×10^{-1}]	5.5×10^{-2}
7	Global PRS	2.30×10^{-2} [-1.36×10^{-2} – 5.96×10^{-2}]	2.17×10^{-1}
	PA	1.50×10^{-2} [-8.9×10^{-3} – 3.89×10^{-2}]	2.19×10^{-1}
	Global PRS x PA	-1.35×10^{-2} [-6.50×10^{-2} – 3.80×10^{-2}]	6.07×10^{-1}
8	AH-PRS	2.65×10^{-2} [-4.29×10^{-2} – 9.58×10^{-2}]	4.54×10^{-1}
	PA	7.7×10^{-3} [-1.91×10^{-2} – 3.45×10^{-2}]	5.73×10^{-1}
	AH-PRS x PA	3.07×10^{-2} [-6.65×10^{-2} – 1.28×10^{-1}]	5.36×10^{-1}
9	C+AH-PRS	3.88×10^{-2} [-1.41×10^{-2} – 9.17×10^{-2}]	1.50×10^{-1}
	PA	1.18×10^{-2} [-1.03×10^{-2} – 3.39×10^{-2}]	2.96×10^{-1}
	C+AH-PRS x PA	-1.18×10^{-2} [-8.54×10^{-2} – 6.18×10^{-2}]	7.53×10^{-1}
10	C-PRS	5.44×10^{-2} [-2.67×10^{-2} – 1.36×10^{-1}]	1.88×10^{-1}
	PA	-4×10^{-4} [-3.26×10^{-2} – 3.18×10^{-2}]	9.80×10^{-1}
	C-PRS x PA	-7.10×10^{-2} [-1.90×10^{-1} – 4.81×10^{-2}]	2.42×10^{-1}
11	E-PRS	-2.01×10^{-2} [-1.44×10^{-2} – 1.04×10^{-2}]	7.50×10^{-1}
	PA	1.19×10^{-2} [-1.61×10^{-2} – 3.98×10^{-2}]	4.04×10^{-1}
	E-PRS x PA	2.14×10^{-2} [-1.52×10^{-1} – 1.95×10^{-1}]	8.08×10^{-2}
12	L-PRS	6.0×10^{-3} [-1.40×10^{-1} – 1.52×10^{-1}]	9.35×10^{-1}
	PA	1.48×10^{-2} [-7.9×10^{-3} – 3.75×10^{-2}]	2.01×10^{-2}
	L-PRS x PA	-2.44×10^{-2} [-2.25×10^{-1} – 1.76×10^{-1}]	8.11×10^{-2}
13	Global PRS	1.20×10^{-2} [-2.33×10^{-2} – 4.73×10^{-2}]	5.06×10^{-1}
	HRF	4.75×10^{-2} [2.48×10^{-2} – 7.02×10^{-2}]	**$<1 \times 10^{-3}$**
	Global PRS x HRF	3.6×10^{-3} [-4.53×10^{-2} – 5.25×10^{-2}]	8.85×10^{-1}
14	AH-PRS	1.03×10^{-2} [-5.51×10^{-2} – 7.56×10^{-2}]	7.58×10^{-1}
	HRF	3.99×10^{-2} [1.46×10^{-2} – 6.53×10^{-2}]	**2×10^{-3}**
	AH-PRS x HRF	5.36×10^{-2} [-3.78×10^{-2} – 1.45×10^{-1}]	2.50×10^{-1}
15	C+AH-PRS	1.73×10^{-2} [-3.21×10^{-2} – 6.67×10^{-2}]	4.92×10^{-1}
	HRF	4.91×10^{-2} [2.80×10^{-2} – 7.02×10^{-2}]	**$<1 \times 10^{-3}$**

	C+AH-PRS x HRF	2.54 x 10^{-2} [4.48 x 10^{-2} – 9.55 x 10^{-2}]	4.78 x 10^{-1}
16	C-PRS	2.91 x 10^{-2} [-4.94 x 10^{-2} – 1.11 x 10^{-1}]	4.67 x 10^{-1}
	HRF	4.51 x 10^{-2} [1.44 x 10^{-2} – 7.59 x 10^{-2}]	**4 x 10^{-3}**
	C-PRS x HRF	-1.70 x 10^{-2} [-1.30 x 10^{-1} – 9.63 x 10^{-2}]	7.68 x 10^{-1}
17	E-PRS	-5.36 x 10^{-2} [-1.73 x 10^{-2} – 6.53 x 10^{-2}]	3.76 x 10^{-1}
	HRF	3.98 x 10^{-2} [1.27 x 10^{-2} – 6.69 x 10^{-2}]	**4 x 10^{-3}**
	E-PRS x HRF	8.66 x 10^{-2} [-7.92 x 10^{-2} – 2.52 x 10^{-1}]	3.06 x 10^{-1}
18	L-PRS	-1.31 x 10^{-2} [-1.49 x 10^{-1} – 1.23 x 10^{-1}]	8.50 x 10^{-1}
	HRF	4.74 x 10^{-2} [2.59 x 10^{-2} – 6.89 x 10^{-2}]	**<1 x 10^{-3}**
	L-PRS x HRF	5.44 x 10^{-2} [-1.37 x 10^{-1} – 2.46 x 10^{-1}]	5.77 x 10^{-1}
19	Global PRS	5.4 x 10^{-3} [-2.36 x 10^{-2} – 3.43 x 10^{-2}]	7.15 x 10^{-1}
	iRORA	3.83 x 10^{-2} [-3 x 10^{-4} – 7.69 x 10^{-2}]	5.2 x 10^{-2}
	Global PRS x iRORA	8.16 x 10^{-2} [5.7 x 10^{-3} – 1.58 x 10^{-1}]	**3.5 x 10^{-2}**
	cRORA	3.94 x 10^{-2} [-9.9 x 10^{-3} – 8.86 x 10^{-2}]	1.17 x 10^{-1}
	Global PRS x cRORA	8.2 x 10^{-3} [-8.75 x 10^{-2} – 1.04 x 10^{-2}]	8.67 x 10^{-1}
20	AH-PRS	1.89 x 10^{-2} [-3.49 x 10^{-2} – 7.27 x 10^{-2}]	4.91 x 10^{-1}
	iRORA	3.27 x 10^{-2} [-9.7 x 10^{-3} – 7.51 x 10^{-2}]	1.31 x 10^{-1}
	AH-PRS x iRORA	1.54 x 10^{-1} [-5.3 x 10^{-3} – 3.13 x 10^{-1}]	5.8 x 10^{-2}
	cRORA	2.53 x 10^{-2} [-3.97 x 10^{-2} – 9.03 x 10^{-2}]	4.45 x 10^{-1}
	AH-PRS x cRORA	5.96 x 10^{-2} [-1.43 x 10^{-2} – 2.62 x 10^{-2}]	5.64 x 10^{-1}
21	C+AH-PRS	1.56 x 10^{-2} [-2.55 x 10^{-2} – 5.66 x 10^{-2}]	4.57 x 10^{-1}
	iRORA	6.40 x 10^{-2} [2.83 x 10^{-2} – 9.96 x 10^{-2}]	**<1 x 10^{-3}**
	C+AH-PRS x iRORA	1.82 x 10^{-1} [6.71 x 10^{-2} – 2.97 x 10^{-1}]	**2 x 10^{-3}**
	cRORA	4.33 x 10^{-2} [4.5 x 10^{-3} – 8.22 x 10^{-2}]	**2.9 x 10^{-2}**
	C+AH-PRS x cRORA	-2.21 x 10^{-2} [-1.63 x 10^{-2} – 1.19 x 10^{-1}]	7.58 x 10^{-1}
22	C-PRS	1.19 x 10^{-2} [-5.36 x 10^{-2} – 7.74 x 10^{-2}]	7.21 x 10^{-1}
	iRORA	1.16 x 10^{-1} [6.39 x 10^{-2} – 1.69 x 10^{-1}]	**<1 x 10^{-3}**
	C-PRS x iRORA	3.13 x 10^{-1} [1.14 x 10^{-1} – 5.11 x 10^{-1}]	**2 x 10^{-3}**
	cRORA	1.99 x 10^{-2} [-3.81 x 10^{-2} – 7.79 x 10^{-2}]	5.01 x 10^{-1}
	C-PRS x cRORA	-1.11 x 10^{-1} [-3.26 x 10^{-1} – 1.04 x 10^{-1}]	3.11 x 10^{-1}
23	E-PRS	-1.67 x 10^{-2} [-1.11 x 10^{-1} – 7.77 x 10^{-2}]	7.28 x 10^{-1}
	iRORA	7.72 x 10^{-2} [2.39 x 10^{-2} – 1.31 x 10^{-1}]	**5 x 10^{-3}**
	E-PRS x iRORA	-1.76 x 10^{-1} [-5.08 x 10^{-1} – 1.56 x 10^{-1}]	2.98 x 10^{-1}
	cRORA	3.17 x 10^{-2} [-2.58 x 10^{-2} – 8.92 x 10^{-2}]	2.8 x 10^{-1}
	E-PRS x cRORA	8.35 x 10^{-2} [-2.70 x 10^{-1} – 4.37 x 10^{-1}]	6.43 x 10^{-1}
24	L-PRS	-2.05 x 10^{-2} [-1.32 x 10^{-1} – 9.10 x 10^{-2}]	7.18 x 10^{-1}
	iRORA	4.72 x 10^{-2} [1.01 x 10^{-2} – 8.43 x 10^{-2}]	**1.3 x 10^{-2}**
	L-PRS x iRORA	2.72 x 10^{-1} [-8.49 x 10^{-2} – 6.28 x 10^{-1}]	1.35 x 10^{-1}
	cRORA	4.02 x 10^{-2} [1.5 x 10^{-3} – 7.9 x 10^{-2}]	**4.2 x 10^{-2}**
	L-PRS x cRORA	8.44 x 10^{-2} [-2.38 x 10^{-1} – 4.07 x 10^{-1}]	6.08 x 10^{-1}

iRORA = Incomplete retinal pigment epithelium and outer retinal atrophy, cRORA = Complete retinal pigment epithelium and outer retinal atrophy, HRF = Hyperreflective foci, PA = Pigmentary abnormalities, RPD = Reticular pseudodrusen, PRS = Polygenic risk score, AH-PRS = ARMS2/HRTA1-PRS, C+AH-PRS = Complement/ARMS2/HTRA1-PRS, C-PRS = Complement-PRS, E-PRS = Extracelular matrix-PRS, L-PRS = Lipid-PRS
